# Rotavirus vaccine administration patterns in Italy: potential impact on vaccine coverage, compliance and adherence

**DOI:** 10.1080/21645515.2020.1816109

**Published:** 2020-09-18

**Authors:** Domenico Martinelli, Francesca Fortunato, Federico Marchetti, Rosa Prato

**Affiliations:** a Dipartimento di Scienze Mediche e Chirurgiche, Università di Foggia, Foggia, Italy; b Medical Department, GSK, Verona, Italy

**Keywords:** Rotavirus, vaccine, Italy, schedule, administration, compliance, adherence, gastro-enteritis, children, pediatric vaccination

## Abstract

Acceptance of rotavirus (RV) vaccination may be impacted by several factors including the feasibility of the full schedule administration within the fixed immunization timelines. The human RV vaccine Rotarix (GSK) and the human bovine reassortant vaccine RotaTeq (Merck & Co.) were developed with different posologies (2 doses vs 3 doses respectively), which have both scientific and technical implications. A non-systematic literature review revealed that, in the Italian context, topics such as time to achieve RV protection in children, number of preventable cases and administration time window, compatibility/ease of inclusion in the national vaccination calendar, potential overlaps with the peak of natural history of intussusception and adherence to posology could be impacted by the RV vaccine posology. Results suggest that a shorter schedule would allow for greater flexibility of use as well as a greater documented ease of inclusion in the vaccination calendar, thereby reducing potential direct healthcare costs.

Rotavirus (RV) is a major cause of acute gastroenteritis throughout the world.^1^ In Europe more than 700,000 medical consultations per year and 87,000 hospitalizations per year for RV illness were estimated before the availability of RV vaccines.^1^ In the end of 2017, in Europe, 14 countries had not considered immunization programs against RV, 5 countries had implemented a partially-funded program and 13 a fully-funded program.^1^

As the vaccine reimbursement pathways vary across European countries, according to the National Health Care regulations, it is very difficult to generate a clear and up-to-date picture of which RV vaccine is used in each European Country, both vaccines being in principle available across Europe.

In Italy, universal vaccination against RV has been included in the Italian National Vaccine Prevention Plan (PNPV) 2017–19,^2^ in an active and free offering throughout the country, based on the regional tender systems to purchase the vaccines at the lowest price offered.

The Italian Ministry of Health has reiterated that starting from those being born in 2017, in addition to mandatory vaccinations recently introduced, RV vaccination is strongly recommended,^3^ as those against pneumococcal and meningococcal diseases, and to be ensured through the so-called ‘2 + 1ʹ immunization calendar of the first year of life.^2^

According to the objectives set out by the Ministry of Health, RV vaccination was expected to reach the following vaccine coverage targets: ≥60% in 2018, ≥75% in 2019 and ≥95% in 2020.^4^

The experience in Sicily, the first Italian region to include an active and free offering of RV vaccination in 2013, documents the difficulty of reaching a high level of coverage, as in 2016 the mean RV vaccination coverage in Sicily reached 45%.^5^ Recent surveys documented the attitude of healthcare providers (HCPs) in Italy in offering RV vaccination to parents. In a survey conducted in Italy in 2015 among family pediatricians, 76.2% of respondents declared that they were convinced on the value of RV vaccination and 57.4% of them stated that they recommended it in their daily practice; however the adherence to RV vaccination was estimated to be <25% due, in the greatest part (60.4%), to poor confidence in the vaccine.^6^ In a more recent web-based survey, more than 85.8% of one thousand family pediatricians spontaneously declared that they would recommend RV vaccination in the future, however more than 40% of them feared that a recommended but not compulsory vaccination might exert a negative impact on family acceptance.^7^

On the other side, although surveys carried out among Italian parents documented significant parental distress due to rotavirus gastroenteritis (RVGE) hospitalization (93.6% reporting high/medium stress), many parents were still unwilling to immunize their babies for RV due to a lack of knowledge of the rotavirus burden of disease and the possibility of preventing RVGE by vaccination.^8^ In such a delicate balance between HCP recommendations and family perception of RVGE burden of disease and vaccine safety, acceptance of RV vaccination may be enhanced by the feasibility of full schedule administration within the fixed immunization calendar.

Two RV vaccines, human RV vaccine (Rotarix; HRV) and human bovine reassortant vaccine (RotaTeq; HBRV) have been approved for clinical use in Europe.^9,10^ Both vaccines have reported good efficacy and favorable safety profiles in preventing RV disease. They differ in posology, with HRV requiring two doses and HBRV three doses.^9,10^

The purpose of this short paper is to discuss the technical and scientific implications arising from the differences in posology of RV vaccines in the Italian context. To prepare this manuscript, a non-systematic, literature review was conducted. References were mainly retrieved from PubMed and Embase with no time limitation but up to September 2019 by combining the following keywords: rotavirus, gastroenteritis, children, pediatric vaccination, rotavirus vaccines, Italy. Other technical or scientific documents were retrieved from Google searches.

The SmPC of HRV states: “The vaccination course consists of two doses. The first dose may be administered from the age of 6 weeks. There should be an interval of at least 4 weeks between doses. The vaccination course should preferably be given before 16 weeks of age, but must be completed by the age of 24 weeks.”^9^

The SmPC of HBRV states: “The vaccination course consists of three doses. The first dose may be administered from the age of 6 weeks and no later than the age of 12 weeks. There should be intervals of at least 4 weeks between doses. It is preferable that the vaccination course of three doses should be completed by the age of 20–22 weeks. If necessary, the third (last) dose may be given up to the age of 32 weeks.”^10^

The time schedules according to the respective SmPC for HRV and HBRV vaccination is presented in Figure 1.

**Figure 1.**
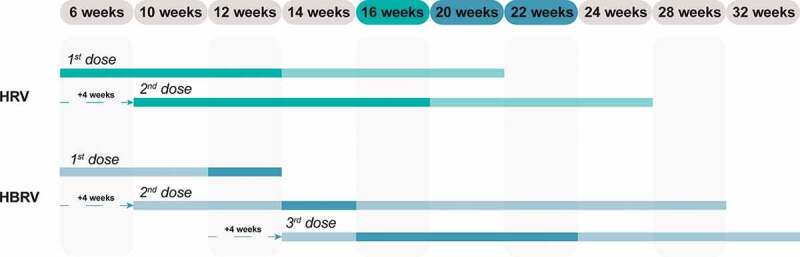
Time schedule for HRV and HBRV vaccination according to the respective SmPC.^9,10^

Legend: Possible (light) and preferred (dark) vaccination schedule for HRV and HBRV vaccination, in green and blue respectively. HBRV: human bovine reassortant vaccine; HRV: human rotavirus vaccine; SmPC: summary of product characteristics.

According to the SmPC, the vaccination course should be completed preferably by 16 weeks of age (about 4 months) for HRV,^9^ and 20–22 weeks (about 5 months) for HBRV.^10^ Efficacy and effectiveness data are based on fully and timely completed courses. Thus, delaying the full protection time could imply an extension of the period of exposure of children to RV and may exert an impact on the general protection of the pediatric population based on the natural history of RVGE. In principle, the risk of developing RVGE increases with increasing age,^11^ and an extended exposure to risk, if considered for an entire birth cohort, may result in a decrease in the number of preventable cases.

Based on data reported in the SmPC of HBRV,^10^ the reason for not administering the first dose beyond 12 weeks of age is likely to be the result of pivotal clinical trials within which this criterion for inclusion was applied. In any case, this indication should be observed in the routine use of the vaccine, precisely for the purpose of replicating the conditions under which the HBRV vaccine was developed. From a practical point of view, this indication, if applied to an entire birth cohort, could affect the number of children that can be vaccinated according to the Italian Immunization Calendar.^2^ Children should begin the vaccination courses at the third month of life, i.e., between 61 and 90 days of age (8–12 weeks of age);^2^ and since the first dose of HBRV should be administered within 12 weeks,^10^ there is an interval of 4 weeks during which the first dose can be administered (Figure 1). If parents delay decision to vaccinate their new-born babies, children may miss their scheduled RV vaccination course.

In accordance with the SmPC of HRV,^9^ the administration of the first dose of HRV is possible up to 20 weeks of age in order to complete the vaccination course by Week 24 as prescribed, thus extending the administration interval of the first dose by an additional 8 weeks compared to the 12 weeks authorized for HBRV (Figure 1).^10^

The extension of the period of exposure of children to the risk of RVGE may result in a lack of prevention of cases requiring hospitalization, thus giving rise to an additional burden for families and direct healthcare costs for the Italian National Health System.^7,8,12^

In principle, by using figures on hospitalizations for RVGE from the Lombardy Region, published by the Italian National Health Institute (ISS) together with the University of Milan,^13^ which reported admissions divided by months of children’s age at the time of admission, some estimates can be reached. In the 1- to 11-month age group (4–44 weeks), the average number of admissions was about 15 per week;^13^ therefore, a 4- to 6-week delay in RV protection may lead to an estimated 60–90 admissions that cannot be prevented due to the incomplete vaccination schedule. If we consider the maximum vaccination periods, i.e., 24 weeks (HRV) or 32 weeks (HBRV), a delay of 8 weeks may lead to an estimate of approximately 120 unprevented hospitalizations due to RVGE per year. Assuming an average cost of €1,478 per hospitalization,^14^ the additional direct health costs could be estimated to approximately €175,000 per year. Based on the PNPV Calendar,^2^ RV vaccination should be administered within the maximum period prescribed by the SmPC for HRV (24 weeks) and HBRV (32 weeks), respectively.^9,10^

Since these are oral vaccines approved for co-administration at the same time as the hexavalent (HX) vaccine (combination of diphtheria, tetanus, acellular pertussis, poliovirus, *Haemophilus influenzae* type B (Hib) and hepatitis B) and pneumococcal conjugate vaccine (PCV), from the public health standpoint, it would be easier to administer the first two doses against RV during the two HX + PCV sessions provided for by the PNPV at the 3^rd^ and 5^th^ month of age (Figure 1). By starting the vaccination course strictly within the scheduled time (at 8 weeks) and planning the administration of the second doses of HX + PCV at Month 5, it is possible to coordinate the co-administration of the two doses of HRV with HX + PCV.

It should be noted that the SmPCs of both HRV and HBRV lack explicit co-administration recommendations with the currently available meningococcal group B pediatric vaccine (MenB). MenB SmPC lacks co-administration data as well.^15^ Recently, the “Calendario per la Vita” Board proposed the co-administration of MenB and RV vaccine.^16^ O’Ryan et al.^17^ reported anecdotal MenB and RV vaccines co-administration during two pivotal clinical trials of MenB vaccine without any interference detected.^17^ In terms of routine clinical practice, it is worth noting that since 2016, the United Kingdom (UK) vaccination calendar has recommended co-administration of the first dose of MenB with the first dose of DTaP (diphtheria, tetanus, and acellular pertussis)/IPV (inactivated polio vaccine)/Hib, PCV and HRV at 2 months of age.^18,19^ As the UK birth cohort in 2018 consisted of more than 657,000 new-borns,^20^ and 90% coverage was reached by the first visit,^19^ there is a huge database of children vaccinated with four vaccines in co-administration. In such a context, no impact has been detected on MenB or RV effectiveness to date.^19^ A systematic review recently published by Pereira et al.^21^ confirmed that no safety issues were observed when HRV was co-administered with MenB in the UK routine immunization program,^22^ and in a small sample of pre-term babies.^23^

Moreover, a devoted visit to administer the remaining dose would be demanding. Assuming 12 minutes for the counseling of parents for each child,^24^ with the presence of two HCPs during the vaccination session (a doctor and an aide/nurse or two aides/nurses and a doctor in the unit) and clinical history assessment and physical examination, vaccine administration and recording (both in the personal vaccination record and healthcare system) to be multiplied by the number of children vaccinated per year, the incremental resources to be invested can be easily perceived. Furthermore, the time devoted by parents or carers to bring the baby to a further vaccination visit should be added and considered as a loss of productivity and an increase in societal costs.

According to the Italian Medicines Agency (AIFA) and the ISS, the incidence of intussusception (IS) in Italy in the period prior to RV vaccination introduction (in 2002–2012) stood at around 19–60 cases per 100,000 in children aged 6–32 weeks.^25^ As shown in Figure 2 the trend for IS risk in Italy reaches its epidemiological peak approximately at the same time as the RV vaccination period.^26^

**Figure 2.**
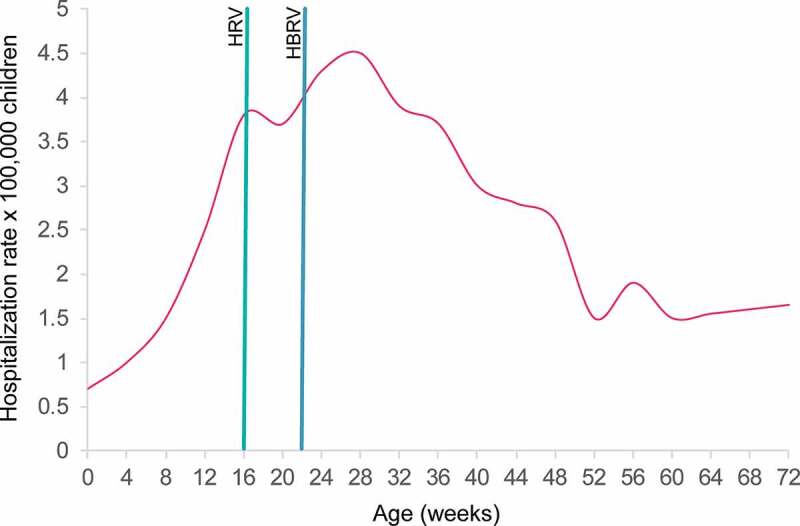
Distribution by infant age (weeks) of hospitalization rates for intussusception in children below 2 years of age in Italy (modified from^26^)

Legend: Preferred timing of vaccination completion according to the HRV and HBRV SmPCs.^9,10^ Green line HRV; blue line HBRV. HRV: human rotavirus vaccine; RVGE: rotavirus gastroenteritis; SmPC: summary of product characteristics.

The SmPCs of both RV vaccines estimate the additional risk of IS after RV vaccination to be in the range of 0–6 cases per 100,000 vaccinations.^9,10^ For both RV vaccines, limited evidence of a lower increase in risk following the second dose is available.^9,10^ Recently, AIFA estimated the risk of additional IS cases after RV vaccination to be 1.5 cases per 100,000 children.^27^ Nevertheless, the Italian scientific community,^16^ and the SmPCs of both RV vaccines,^9,10^ recommend completing the RV vaccination as early as possible to reduce the risk of an IS episode close to post-vaccination period of time (Figure 2). Furthermore, the early completion of RV vaccination schedule would contribute to a reduction of RVGE in newborns, which was reported to be one of the factors closely associated with IS (odd ratio 11.55, 95% confidence interval: 3.23, 41.23, *P* < .001) in a case-control study conducted in Italy.^28^

It is a common experience that the total number of doses needed to complete a vaccination course affects the likelihood of completing the course (compliance) within the time frame indicated by the SmPC (adherence).^29-34^

Measuring compliance and adherence for the HRV and HBRV vaccines requires accurate data and can therefore only be carried out effectively in highly organized contexts. Some studies conducted in the United States of America (USA), where a ‘3 + 1ʹ vaccination schedule is used for the first year of life, among patients under different health insurance systems are available in the literature.^29-32^ During the launch of RV vaccination in the USA in 2006, some pediatricians set themselves the objective of measuring adherence with vaccination in the presence of criteria they claimed to be restrictive, i.e., before 12 weeks of age for the first vaccine dose.^29^ During the first 6 months after RV vaccination implementation, 770 (19.7%) of the 3,912 children vaccinated with HBRV received their first dose outside the prescribed time limits. According to the authors, the age limit below 12 weeks of age resulted in around 23% reduction in subjects who could be vaccinated in the whole study period, with a reduction between 16% and 30.5% considering year by year (30.5% in 2001 vs 16% in 2005).^29^

A study conducted in 2009 in the USA among children under private care showed that the use of HRV resulted in a higher compliance rate compared to HBRV (91.0% vs 83.4%) and a lower age at the completion of the RV vaccination course.^30^ Another study conducted within the Medicaid system evaluated compliance rates and adherence to the vaccination schedule in a cohort of 673,956 children under the age of one year, comparing the two approved RV vaccines, HRV and HBRV.^31^ When stratified by type of vaccine, children who received HRV as their first dose had a significantly higher adherence rate than those who received HBRV as their first dose (65.2% vs 31.3%; *P* < .0001).^31^ The analysis showed similar results also when considering non-adherence by dose: both the 1^st^ and the 2^nd^ dose had a higher rate of noncompliance for the HBRV cohort than for the HRV cohort (20.1% vs 1.9% and 49.2% vs 34.8%).^31^

The same study evaluated the compliance to the vaccination schedule, defined as the administration of 2 doses of HRV or 3 doses of HBRV to each child. The compliance rate was 1.4 times greater (65.3% vs 46.4%) for schedule completion of HRV compared to HBRV (Table 1).^31^

**Table 1.** Compliance to vaccination schedule (% per dose).^31^

Vaccine	Dose 1	Dose 2	Dose 3
HRV	100	65.3	/
HBRV	100	79.1	46.4

HBRV: human bovine reassortant vaccine; HRV: human rotavirus vaccine.

In a retrospective study also conducted in the USA but in a population of children with healthcare insurance, a cohort of 162,614 children scheduled to be vaccinated according to the HRV or HBRV SmPCs was evaluated.^32^ Of the children vaccinated with HBRV, 24% did not complete the schedule and 76% completed the course, but only 54% completed it according to the established dosing schedule. Of the children vaccinated with HRV, 15% did not complete the schedule, 85% completed the HRV course, and 69% completed the schedule on time.^32^ The authors pointed out that the children who did not complete the vaccination course were predominantly those who started vaccination with a few weeks delay, and therefore more attention should be paid to this group.^32^

In Mexico, after a national switch from HRV to HBRV, a significant decline in vaccination coverage at national level (75.6% vs 61.0%, *P* < .001) was recorded despite institutional efforts in vaccination implementation. The RV vaccination compliance also decreased, as only 71.1% of HBRV primed infants completed the full series compared to 93.7% (*P* < .001) of HRV primed infants in previous years.^33^

In Belgium, a follow-up study carried out in 2007–2012 showed that on average, 85.4% (range: 80.7–88.2%) of eligible infants were vaccinated against RV: 88.0% (range: 80.5–99.0%) of them with HRV and 12.0% (range: 9.5–19.5%) with HBRV. In this time frame, the number of children who did not complete their vaccination course decreased from 10.8% to 7.9%. However, within these figures, this non-compliance originates mainly from HBRV compared to HRV (17.3% vs 6.8% in 2012).^34^

Finally, very recently, a local experience in Italy carried out in 2018–2019 assessed RV vaccination coverage and compliance.^35^ The authors reported a significantly higher RV vaccination coverage with HRV than HBRV (43.9% vs 7.07%, *P* < .001 in 2019). In terms of compliance, 83.18% of HRV vaccinees completed the schedule in 2019 as compared to 75.00% (*p* < .001) of HBRV recipients.^35^

The recent implementation of universal RV mass vaccination in Italy poses some operational challenges in order to achieve the targeted vaccination coverage. RV vaccination acceptance by the families needs convenient counseling from the HCPs, a clear understanding of the RV burden of disease, and trust in the RV vaccines safety, tolerability and efficacy; furthermore, it has to be well fitted into the vaccination visit series. The posology of RV vaccines results in a series of technical and scientific differences that may affect aspects such as the time to achieve RV protection in children and a narrow administration time window for HBRV (since an interval of 4 weeks is needed between doses),^9,10^ potentially leading to an increased number of unprevented hospitalizations with additional direct health costs per year.^11,13,16^ Other implications may arise from: i) the co-administration of HRV with HX and PCV in a ‘2 + 1ʹ country schedule, turning out to be less complex for HRV than HBRV; ii) the completion of the vaccination course in a shorter time, thereby reducing the RV vaccination time overlap with the epidemiological peak of IS;^25,26^ iii) a better posology adherence with a shorter schedule, as fully documented in studies from the USA, Mexico, Belgium and Italy.^29–35^ Finally, a shorter RV vaccine schedule may also reduce the financial burden for both the healthcare system and the families of the vaccinated infants (health and administrative staff, economic resources, time and family discomfort).

In the era of universal RV mass vaccination implementation at national level in Italy, significant efforts are needed to reach the vaccination coverage set by the Ministry of Health. Further to the engagement of the HCPs and the education of families with infants on the value of preventing RVGE and the safety and effectiveness of RV vaccines, in the light of the deeply discussed international data, in the Authors’ opinion, a shorter dosing schedule would allow for greater dosing flexibility as well as a documented ease of inclusion in the established vaccination calendar. Moreover, a shorter vaccination schedule may free up resources that could be reinvested in achieving higher vaccination coverage for the RV vaccine or for other vaccines included in the calendar.

Figure 3 elaborates on the findings of this review in a form that could be shared with patients by HCPs.

**Figure 3.**
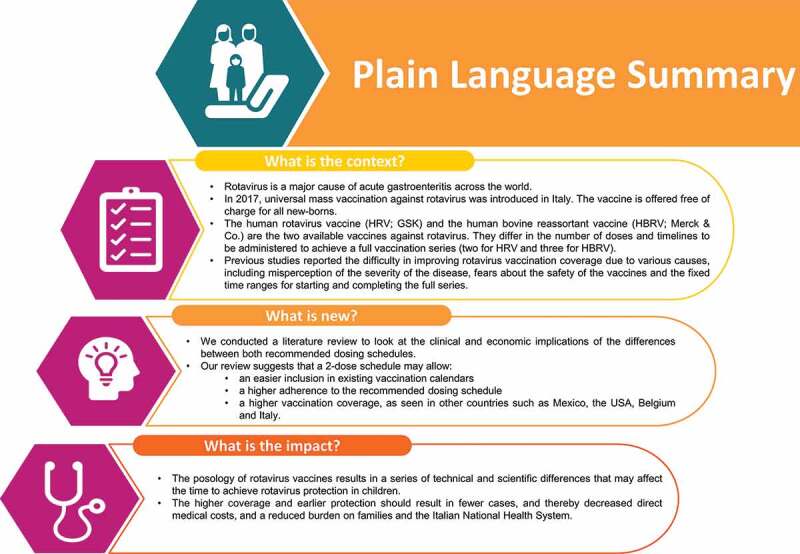
Plain language summary

## Data Availability

Data sharing is not applicable to this article as no new data were created or analyzed in this research.

## Trademark statement

RotaTeq is a trademark owned by or licensed to Merck & Co, USA.

Rotarix is a trademark owned by or licensed to the GSK group of companies.

## References

[1] Poelaert D, Pereira P, Gardner R, Standaert B, Benninghoff B. A review of recommendations for rotavirus vaccination in Europe: arguments for change. Vaccine. 2018;36(17):2243–53. doi:10.1016/j.vaccine.2018.02.080. PMID:29576308. 29576308

[2] Ministero della Salute. Piano Nazionale Prevenzione Vaccinale: PNPV 2017–2019. In Gazzetta Ufficiale della Repubblica Italiana; 2017. . [accessed 2020 127]. http://www.trovanorme.salute.gov.it/norme/renderPdf.spring?seriegu=SG&datagu=18/02/2017&redaz=17A01195&artp=1&art=1&subart=1&subart1=10&vers=1&prog=001.

[3] Ministero della Salute. CIRCOLARE del Ministero della Salute n. 25146 Circolare recante prime indicazioni operative riguardanti il comma 1-quater, art. 1 del decreto-legge n. 73 del 7 giugno 2017, convertito con modificazioni dalla legge 31 luglio 2017, n. 119, recante “Disposizioni urgenti in materia di prevenzione vaccinale, di malattie infettive e di controversie relative alla somministrazione di farmaci” (17G00132) (GU Serie Generale n. 182 del 05-08-2017); 2017 [accessed 2020 127]. http://www.trovanorme.salute.gov.it/norme/renderNormsanPdf?anno=2017&codLeg=60284&parte=1%20&serie=null.

[4] Ministero della Salute. CIRCOLARE del Ministero della Salute n. 7903 Aspetti operativi per la piena e uniforme implementazione del nuovo PNPV 2017–2019 e del relativo Calendario Vaccinale; 2017 [accessed 2020 127]. http://www.trovanorme.salute.gov.it/norme/renderNormsanPdf?anno=2017&codLeg=58583&parte=1%20&serie=null.

[5] Costantino C, Restivo V, Tramuto F, Casuccio A, Vitale F. Universal rotavirus vaccination program in Sicily: reduction in health burden and cost despite low vaccination coverage. Hum Vaccin Immunother. 2018;14(9):2297–302. doi:10.1080/21645515.2018.1471306. PMID: 29757707. 29757707 PMC6183134

[6] Mita V, Arigliani M, Zaratti L, Arigliani R, Franco E. Italian physicians’ opinions on rotavirus vaccine implementation. Pathogens. 2017;6(4):56. doi:10.3390/pathogens6040056. PMID:29099756. PMC5750580 29099756

[7] Marchetti F, Conforti G. Seconda indagine conoscitiva sull’opinione dei pediatri di famiglia italiani in merito alla vaccinazione contro i rotavirus. Societa Italiana di Igiene, Medicina Preventiva e Sanita Pubblica - 51st Congresso Nazionale. Riva del Garda (Italy); 2018. p. 225.

[8] Marchetti F, Vetter V, Conforti G, Esposito S, Bonanni P. Parents’ insights after pediatric hospitalization due to rotavirus gastroenteritis in Italy. Hum Vaccin Immunother. 2017;13(9):2155–59. doi:10.1080/21645515.2017.1336271. PMID:28609219. 28609219 PMC5612036

[9] European Medicines Agency. Rotarix: EPAR - product information; 2019 [Date of last update 2019 405]. https://www.ema.europa.eu/en/documents/product-information/rotarix-epar-product-information_en.pdf.

[10] European Medicines Agency. RotaTeq: EPAR - product Information; 2018 [Date of last update 2018 1207]. https://www.ema.europa.eu/en/documents/product-information/rotateq-epar-product-information_en.pdf.

[11] Velazquez FR, Matson DO, Calva JJ, Guerrero L, Morrow AL, Carter-Campbell S, Glass RI, Estes MK, Pickering LK, Ruiz-Palacios GM. Rotavirus infection in infants as protection against subsequent infections. N Engl J Med. 1996;335(14):1022–28. doi:10.1056/NEJM199610033351404. PMID:8793926. 8793926

[12] Napolitano F, Ali Adou A, Vastola A, Angelillo IF. Rotavirus infection and vaccination: knowledge, beliefs, and behaviors among parents in Italy. Int J Environ Res Public Health. 2019;16(10):1807. doi:10.3390/ijerph16101807. PMID:31117274. PMC6571979 31117274

[13] Pellegrinelli L, Bubba L, Primache V, Chiaramonte I, Ruggeri FM, Fiore L, Binda S. Burden of pediatrics hospitalizations associated with Rotavirus gastroenteritis in Lombardy (Northern Italy) before immunization program. Ann Ist Super Sanita. 2015;51(4):346–51. doi:10.4415/ANN_15_04_16. PMID:26783223. 26783223

[14] Panatto D, Amicizia D, Ansaldi F, Marocco A, Marchetti F, Bamfi F, Giacchino R, Tacchella A, Del Buono S, Gasparini R. Burden of rotavirus disease and cost-effectiveness of universal vaccination in the Province of Genoa (Northern Italy). Vaccine. 2009;27(25–26):3450–53. doi:10.1016/j.vaccine.2009.01.054. PMID:19200850. 19200850

[15] European Medicines Agency. Bexsero: EPAR - product information; 2018 [Date of last update 2018 711]. https://www.ema.europa.eu/en/documents/product-information/bexsero-epar-product-information_en.pdf.

[16] Societa Italiena di Pediatria. Calendario per la Vita, 4° edizione 2019; 2019 [accessed 2020 127]. https://www.sip.it/wp-content/uploads/2019/07/Calendario-vaccinale-per-la-Vita-2019.pdf.

[17] O’Ryan M, Stoddard J, Toneatto D, Wassil J, Dull PM. A multi-component meningococcal serogroup B vaccine (4CMenB): the clinical development program. Drugs. 2014;74(1):15–30. doi:10.1007/s40265-013-0155-7. PMID:24338083. 24338083 PMC3890039

[18] Public Health England. The complete routine immunisation schedule from summer 2016. [born on or before 2017 731]; 2016 [accessed 2020 127]. https://assets.publishing.service.gov.uk/government/uploads/system/uploads/attachment_data/file/533829/9699_PHE_2016_Complete_Immunisation_Schedule_SUMMER16_A4_16.pdf.

[19] Screening & Immunisations Team ND. Childhood vaccination coverage statistics, England 2017–18, national statistics; 2018 [accessed 2020 127]. https://files.digital.nhs.uk/55/D9C4C2/child-vacc-stat-eng-2017-18-report.pdf.

[20] Office for National Statistics. Births in England and Wales: 2018; 2019 [accessed 2020 211]. https://www.ons.gov.uk/peoplepopulationandcommunity/birthsdeathsandmarriages/livebirths/bulletins/birthsummarytablesenglandandwales/2018.

[21] Pereira P, Benninghoff B, Moerman L. Systematic literature review on the safety and immunogenicity of rotavirus vaccines when co-administered with meningococcal vaccines. Hum Vaccin Immunother. 2020:1–12. doi:10.1080/21645515.2020.1739485.PMID: 32298219. PMC7746238 32298219

[22] Bryan P, Seabroke S, Wong J, Donegan K, Webb E, Goldsmith C, Vipond C, Feavers I. Safety of multicomponent meningococcal group B vaccine (4CMenB) in routine infant immunisation in the UK: a prospective surveillance study. Lancet Child Adolesc Health. 2018;2(6):395–403. doi:10.1016/s2352-4642(18)30103-2. PMID: 30169281. 30169281

[23] Sadarangani M, Barlow S, Anthony M, Pollard AJ. Four component meningococcal capsular group B vaccine in preterm infants. J Pediatric Infect Dis Soc. 2017;6(3):309–10. doi:10.1093/jpids/pix013. PMID: 28369464. 28369464

[24] ASL Mantova. Il Manuale delle Vaccinazioni; 2015 [accessed 2020 127]. https://www.aslmn.net/docs_file/Manuale_delle_Vaccinazioni.pdf.

[25] Trotta F, Da Cas R, Bella A, Santuccio C, Salmaso S. Intussusception hospitalizations incidence in the pediatric population in Italy: a nationwide cross-sectional study. Ital J Pediatr. 2016;42:89. doi:10.1186/s13052-016-0298-8. PMID:27677340. 27677340 PMC5039877

[26] Mattei A, Fiasca F, Mazzei M, Sbarbati M. Unparalleled patterns of intussusception and rotavirus gastroenteritis hospitalization rates among children younger than six years in Italy. Ann Ig. 2017;29:38–45. doi:10.7416/ai.2017.2130. PMID:28067936. 28067936

[27] Agenzia Italiana del Farmaco. Rapporto vaccini 2018; 2018 [accessed 2020 127]. https://www.aifa.gov.it/documents/20142/241056/Rapporto+Vaccini+2018.pdf/62975a0e-a836-da32-f7b9-1e39196374dd.

[28] Restivo V, Costantino C, Giorgianni G, Cuccia M, Tramuto F, Corsello G, Casuccio A, Vitale F. Case-control study on intestinal intussusception: implications for anti-rotavirus vaccination. Expert Rev Vaccines. 2018;17(12):1135–41. doi:10.1080/14760584.2018.1546122. PMID: 30407079. 30407079

[29] Daskalaki I, Spain CV, Long SS, Watson B. Implementation of rotavirus immunization in Philadelphia, Pennsylvania: high levels of vaccine ineligibility and off-label use. Pediatrics. 2008;122(1):e33–38. doi:10.1542/peds.2007-2464. PMID:18595974. 18595974

[30] Krishnarajah G, Davis EJ, Fan Y, Standaert BA, Buikema AR. Rotavirus vaccine series completion and adherence to vaccination schedules among infants in managed care in the United States. Vaccine. 2012;30(24):3717–22. doi:10.1016/j.vaccine.2011.12.077. PMID:22214886. 22214886

[31] Krishnarajah G, Landsman-Blumberg P, Eynullayeva E. Rotavirus vaccination compliance and completion in a Medicaid infant population. Vaccine. 2015;33(3):479–86. doi:10.1016/j.vaccine.2014.06.059. PMID:24962753. 24962753

[32] Eisenberg DF, Gu T, Krishnarajah G. Adherence to rotavirus vaccination quality measures in a commercially insured population. Hum Vaccin Immunother. 2013;9(2):389–97. doi:10.4161/hv.22877. PMID:23291933. 23291933 PMC3859762

[33] Luna-Casas G, Juliao P, Carreno-Manjarrez R, Castaneda-Prado A, Cervantes-Apolinar MY, Navarro-Rodriguez R, Sanchez-Gonzalez G, Cortes-Alcala R, DeAntonio R. Vaccine coverage and compliance in Mexico with the two-dose and three-dose rotavirus vaccines. Hum Vaccin Immunother. 2019;15(6):1251–59. doi:10.1080/21645515.2018.1540827. PMID:30380975. 30380975 PMC6783135

[34] Sabbe M, Berger N, Blommaert A, Ogunjimi B, Grammens T, Callens M, Van Herck K, Beutels P, Van Damme P, Bilcke J. Sustained low rotavirus activity and hospitalisation rates in the post-vaccination era in Belgium, 2007 to 2014. Euro Surveill. 2016;21(27). doi:10.2807/1560-7917.ES.2016.21.27.30273. PMID: 27418466. 27418466

[35] Aquilani S, Dari S, Fiasca F. Assessing rotavirus vaccination coverage and compliance after two years of local experience in Italy. Ann Ig. 2020;32(4):433–35. doi:10.7416/ai.2020.2367.PMID: 32744302. 32744302

